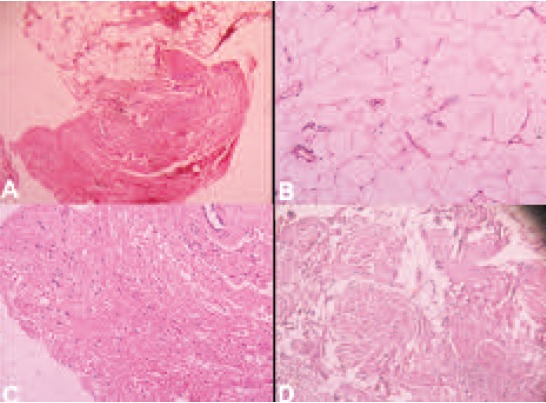# Concurrent Klippel-Feil Anomaly, Tethering and Dermoid Cyst Misinterpreted as Pott disease: A Case Report

**DOI:** 10.5704/MOJ.1403.002

**Published:** 2014-03

**Authors:** A Agrawal, T Gopalkrishnaiah, V Shanthi, BA Ramakrishna

**Affiliations:** Department of Neurosurgery, Narayana Medical College Hospital, Nellore, India; Department of Orthopaedics, Narayana Dental College Hospital, Nellore, India; Department of Pathology, Narayana Medical College Hospital, Nellore, India; Department of Pathology, Narayana Medical College Hospital, Nellore, India

## Abstract

Klippel-Feil syndrome (KFS) is characterized by the failure in segmentation of two or more vertebrae due to an abnormal division of the mesodermal somites and has been reported to be associated with cardiac and central nervous system anomalies. We report a rare occurrence of concurrent Klippel-Feil anomaly, tethering and dermoid cyst of dorsal spine masquerading Potts’ disease in an eighteen years old female. In rare instances the KFS can be associated with intracranial or spinal tumors, most frequently dermoid or epidermoid cysts.

**Key Words:**

Congenital spinal malformation, Klippel-Feil syndrome, dermal sinus, dermoid cysts

## Introduction

Klippel-Feil syndrome (KFS) is characterized by the failure in segmentation of two or more vertebrae due to an abnormal division of the mesodermal somites ^1, 2^ and has been reported to be associated with cardiac and central nervous system anomalies^3^. In rare instances the KFS can be associated with intracranial or spinal tumors, most frequently dermoid or epidermoid cysts^1, 4, 5^. We report the rare occurrence of concurrent Klippel-Feil anomaly associated with intraspinal lesion, tethering and dermoid cyst misinterpreted as Potts’ disease.

## CASE REPORT

**Fig. 3: Intra-operative photograph showing fused spinous process of D8 and D9 vertebral bodies.**
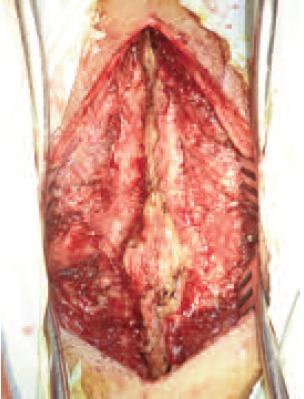


An 18- year old female presented with a history of progressive weakness of both lower limbs with difficulty in walking. She had been bedridden for one month. She had hesitancy of micturition, but no urinary retention. There was no history of upper limb weakness. There was no history of contact with tuberculosis. General and systemic examinations were unremarkable. Higher mental functions and cranial nerves were normal. Motor and sensory functions in upper limbs were normal. There was increased tone in both the lower limbs. Sensations were decreased to all modalities below D8 level. Power was grade II/V in the lower limbs. Bilateral knee and ankle jerks were exaggerated and the plantar response was extensor. AP and lateral radiographs of the dorsal spine showed reduced disc space between D8 and 9 vertebral bodies and scalloping of the posterior margins (Figure 1) and (Figure 2). MRI dorsal spine showed a well-defined intraspinal mass lesion extending from, D8 to D10 vertebral levels, compressing the spinal cord. The lesion was hypo-intense on T1W images and mildly hyperintense on T2W images (Figure 2). Spinal tuberculosis was suspected from the initial findings. At operation through a posterior midline spinal approach, fusion of D9-D10 spinous process was noted. (Figure 3). D8 to D10 laminectomy was performed. There was a thin fibrous tract connecting the bone and dura, and continuous intradurally over the lesion. There was pale yellow ill-defined lesion displacing the spinal cord to the right, connected with the fibrous band to the dura. The lesion was partly excised, and below and thick yellowish non-purulent material came out from below and medial aspects of the lesion. This fluid was cultured and found to be sterile and negative for AFB on Ziehl–Neelsen staining. After surgery, the imaging findings were retrospectively reviewed and narrowing of the neural foramina was identified. Although not very apparent there was fusion of the spinous processes on plain radiographs and MRI. Histopathology of the excised tissue revealed lobules of mature adipocytes with adjacent glial tissue with congested blood vessels and smooth muscle bundles. Day after the surgery, patient's power in the lower limbs improved to Grade 3/5. Urethral catheter was removed on the 4th postoperative day, as the patient regained bladder control. The patient was able to walk with support on the 15th postoperative day and was discharged. At one year follow up the patient was doing well.

## Discussion

An array of congenital central nervous system abnormalities have been described in patients with Kilppel-Feil syndrome (i.e. meningocele, spinal dysraphism, possibly spinal cord malformation and dermoid cysts at various levels) which develop - during the same intra-uterine period as the development of the somites^2, 5^. Congenital fusion of the vertebrae in Klippel-Feil syndrome is due to failure of normal segmentation of the cervical somites during the third to eighth week of gestation^4^. It has been suggested that a disturbance in the mesoderm before the fourth week of gestation might play an important part in the causation of these anomalies^1^. A shortening of the spine because of a reduction or fusion in the number of somites may result in altered tissue tension, which could lead to entrapment of dermal elements^4^. The classic clinical triad of a short neck, low hairline, and limitation of movement of the neck which is seen in approximately 52% of patients with KFS4 can be absent in patients where there is no involvement of the cervical spine. Although MRI is the best investigation to show the extent of intra-spinal lesions but supplementing with plain radiograph and CT scan provide details of the bony pathology. A total surgical resection is the mainstay of treatment of these lesions^5^. In the current case, initial radiographs showed decreased disc space, MRI showed apparently smaller vertebrae, the findings consistent with an intraspinal cystic lesion, leading to misdiagnosis as spinal tuberculosis; however a definitive diagnosis was made after histopatholigcal examination.

**Fig. 1: Plain radiography of dorsal spine AP and lateral view showing reduced disc space between D8 and 9 vertebral bodies and scalloping of the posterior margins. Narrowing of the neural foramina and fusion of the posterior elements is noted.**
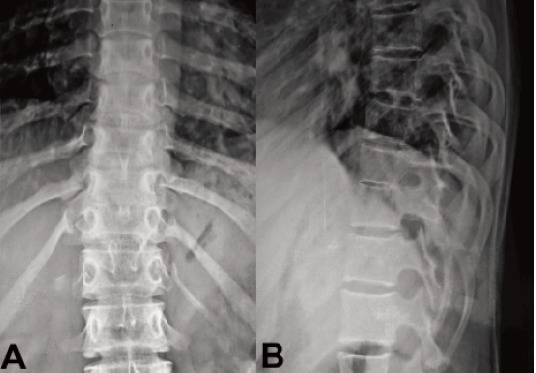


**Fig. 2: MRI dorsal spine showing an intraspinal well defined mass lesion extending from D8 to D10 vertebral levels and compressing the spinal cord. The lesion was hypo intense on T1W images and mildly hyper-intense on T2W images.**
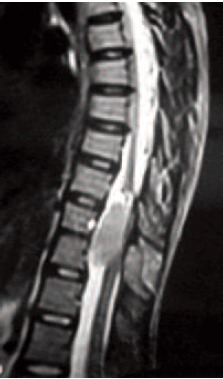


**Fig. 4: (A) Lobules of mature adipocytes with adjacent glial tissue (left bottom) (H&E, x50), (B) lobules of mature adipocytes with congested blood vessels (H&E, x100), (C) glial tissue with fibrillary background (H&E, x100) and (D) smooth muscle bundles (H&E, x 100).**